# Fast decision mechanism for ternary tree partitioning in VVC intra coding

**DOI:** 10.1371/journal.pone.0341803

**Published:** 2026-02-06

**Authors:** Jiamin Sun, Zhongjie Zhu, Renwei Tu, Zhibo Xie

**Affiliations:** 1 Key Laboratory of Industrial Vision and Industrial Intelligence, Zhejiang Wanli University, Ningbo, China; 2 Faculty of Information Science and Engineering, Ocean University of China, Qingdao, China; Aalto University, FINLAND

## Abstract

In Versatile Video Coding (VVC), the partition patterns for coding units (CUs) have significant impact on the encoding efficiency. Determining the optimal CU partition is particularly time-consuming due to the calculation and comparison of rate-distortion costs for all possible partition patterns, especially during the ternary tree (TT) partitioning in intra coding. In this paper, a fast decision mechanism is proposed for TT partitioning based on image feature analysis to skip the complex rate-distortion calculation. Firstly, the correlation between the image structural features and the TT partition patterns is investigated based on experimental analysis and the most relevant features are selected for the subsequent prediction of optimal TT partition patterns. Secondly, we devise an efficient scheme for representing and extracting the selected features, further optimizing the extraction process to minimize computational complexity. Comprehensive datasets for partition pattern prediction are constructed based on these refined features. Finally, these datasets serve as the foundation for training and optimizing a predictive model, which is designed to achieve an optimal trade-off between prediction accuracy and model complexity. The predictive model is seamlessly incorporated into the VVC Test Model (VTM), facilitating efficient feature extraction prior to the Rate-Distortion Optimization (RDO) process for intra prediction and optimal partition pattern selection. By leveraging the prediction results, the model effectively determines whether TT partitioning can be bypassed, thereby streamlining the decision-making process and enhancing overall coding efficiency. Experimental results demonstrate that in comprehensive performance evaluations of time-saving metrics and Bjøntegaard Delta Bit Rate (BDBR), the proposed mechanism significantly outperforms existing lightweight neural network algorithms. Our decision mechanism effectively preserves coding quality while substantially accelerating the video coding process.

## 1 Introduction

With the widespread application of high-definition and high dynamic range videos, the requirements for video coding performance are also increasing. Accordingly, the joint video expert team developed versatile video coding (VVC), a next generation video coding standard. VVC supports high-definition, ultra-high-definition, and high dynamic range videos, as well as virtual reality videos, 360° panoramic videos, and screen content [1]. Compared to high efficiency video coding (HEVC) [2,3], VVC can reduce bitrate by about 50% while maintaining the same video quality [4], making it a key technology for meeting current and future video coding needs.

VVC and HEVC are block-based coding standards and their compression performance is closely related to the partitioning structure of the coding unit (CU). Unlike HEVC, which uses a quad-tree (QT) pattern to partition the CU, VVC has significantly optimized CU partition patterns [5], introducing a combination of a binary tree (BT) and a ternary tree (TT) block partitioning, termed the multi-type tree (MTT) structure-within a structure called a quad-tree plus multi-type tree (QTMT) structure. This QTMT structure allows for rectangular block partitioning, enhancing the flexibility of the CU partitioning to better adapt to various local video features, thereby improving coding efficiency and quality. In the VVC test model (VTM), the optimal partition pattern can be determined using rate distortion optimization (RDO). The rate distortion (RD) cost of all partition patterns in the candidate list is calculated, and the pattern with the lowest RD cost is selected as the optimal partition pattern. However, with an increase in the partition patterns, the number of candidate CUs and candidate patterns increases sharply, making the partition structure more complex, thereby increasing the coding complexity.

The process of VVC coding involves intra and inter coding, each addressing a different type of frame within the video. Intra coding [6] is specifically designed for coding intra frames and plays a crucial role in ensuring video quality and coding independence and improving robustness. By contrast, inter coding handles predictive frames and bidirectional predictive frames, offering higher compression efficiency than intra coding. However, inter coding relies on the intra-frames processed by intra coding as references. Thus, enhancing the efficiency of intra coding is vital for optimizing the overall video coding process.

To accelerate the decision process for intra coding partition patterns, shorten video coding time, and reduce coding complexity, scholars have conducted a series of studies on fast intra coding for intra frames. These studies have included methods based on the gradient of the CU to be partitioned, traditional machine learning models, and deep learning models. The first is the method of determined the partition patterns based on the gradient of the block to be partitioned. Chen et al. [7] selected a QT partition based on the gradient extracted using a Sobel operator. However, this method was only applicable only to CUs with a size of 32 × 32. Liu et al. [8] determined whether to skip the current partition pattern directly based on the directional gradient difference between the subdivided blocks. However, there is a lack of discussion regarding the defined thresholds. Cui et al. [9] estimated the likelihood of BT and TT partitioning in both the horizontal and vertical directions, thereby effectively skipping irrelevant partition patterns. Their approach involves computing directional gradients in four orientations for each block to be partitioned, which leads to relatively high computational complexity.

Compared with the aforementioned methods, methods based on traditional machine learning make better use of the correlation between the features of the CU and the optimal partition pattern to directly predict the partition patterns. He et al. [10] classified CUs into three types, that is simple, ambiguous, and complex CUs. Consequently, for different types of CUs, different random forest classifiers were trained to predict the optimal partition patterns; however these methods were prone to overfitting. Wu et al. [11] used support vector machines and texture information to predict CU partitions. Classifiers were trained for different CU sizes, with each classifier having its own threshold; however the large number of classifiers made the implementation complex. Yang et al. [12] proposed a fast decision framework based on a gradient descent search to determine QTMT partitioning. Saldanha et al. [13] trained five classifiers based on a light gradient boosting machine (LightGBM) model to predict five CU partition patterns according to texture, coding, and contextual information. Similar to [11], each classifier had a different threshold; however the Bjontegaard delta bit rate (BDBR) increased by 2.43%.

Although traditional machine learning models have lower complexity, their prediction accuracy is not high, and they can be prone to overfitting. Thus, with advances in deep learning, numerous methods based on convolutional neural networks (CNNs) have emerged. Tang et al. [14] proposed an adaptive CU segmentation CNN that effectively used pooling layers for different CU shapes; however it reduced the coding time by just 33.41%. Tissier et al. [15] trained a CNN to predict the probability vector for each 64 × 64 CU. The encoder then used this probability vector to skip the unlikely partitions. However, because their database consisted of 4K images primarily, their performance at lower resolutions was not as good as that at higher resolutions. Wu et al. [16] introduced a hierarchical grid full-convolution network that could obtain the entire partition information of the current CU and sub-CUs in a single inference; however the model only predicted for 32 × 32 CUs and their sub-blocks. Cao et al. [17] analyzed the relationships between the first round of CUs and subsequent rounds, identifying and preemptively terminating unnecessary partition types, and achieving adaptive threshold adjustments to develop a hierarchical pruning algorithm; however, the coding time was only reduced by approximately 20%. Li et al. [18] established a large-scale database and proposed a multistage exit CNN model with an early exit mechanism to determine CU partitions. However, numerous factors still need to be considered, such as the accurate prediction of CU partition patterns and the efficiency of neural network utilization. Zan et al. [19] used U-Net as the basic network framework and developed a quantization parameter (QP) fusion network to adjust the impact of the QP on partition outcomes. Tissier et al. [20] used a CNN to describe the probability vector of each 4 × 4 boundary partition to predict the spatial features to establish a public VVC partition dataset. Based on the prediction results, only the N most likely partitions were calculated for the RD cost using the encoder. Park et al. [21] developed two types of features and proposed a lightweight neural network (LNN) model to determine whether to terminate a TT partition. Although the LNN model had low complexity, it exhibited a low prediction accuracy of just 75%. Compared to traditional machine learning models, deep learning models offer higher prediction accuracy; however, this is achieved through more complex network structures, resulting in longer training and prediction times, without significantly reducing the coding time.

To enhance video coding efficiency and reduce the computational complexity of the rate-distortion optimization process, this paper proposes a structural feature analysis-based method to balance model accuracy and complexity. In the RDO process, computational cost increases with partition depth and the number of sub-blocks after CU partitioning. In the QTMT framework, quadtree partitioning, which generates the most sub-blocks, is suitable for shallow partitions but cannot be used after multi-type tree partitioning. Conversely, TT partitioning, producing more sub-blocks, is better for deeper partitions. Accordingly, this work introduces a fast TT partition decision mechanism for VVC intra coding, which effectively skips unnecessary TT partition evaluations and optimizes the coding process. The main contributions of this study are as follows:

(1) We investigate the relationship between optimal CU partition patterns and image structural features. We extract salient CU features, and perform feature selection based on their statistical significance. Building upon the observation that CU partition boundaries frequently coincide with texture edges, we leverage texture feature analysis to facilitate accurate prediction of partition patterns.
(2) To accurately capture the impact of texture variations on luminance information, we construct a dataset tailored for predicting TT partition patterns. The pixel luminance gradient is computed to represent the texture variations within video frames. Subsequently, these gradient-based features are refined by integrating the structural features of TT partitions, thereby facilitating efficient feature extraction.
(3) We develop and optimize a predictive model, which is integrated into the VTM 4.0 framework, to forecast optimal partitioning patterns. This approach enables us to bypass the computationally expensive RD cost calculations when predictions indicate that certain TT partitioning patterns are unlikely to yield optimal results. Consequently, this significantly reduces encoding time.

## 2 Motivation and algorithm

**Fig 1:**
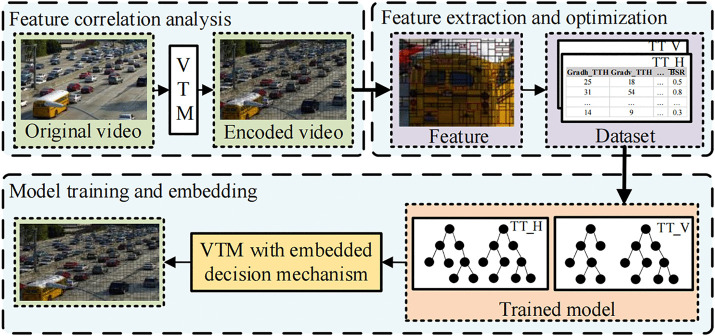
Fast decision mechanism for TT partition.

As illustrated in Fig 1, the proposed decision mechanism is divided into three main modules, that is, the feature correlation analysis, feature extraction and optimization, and model training and embedding modules. The first module visualizes the CU partition results of the VTM coding common test conditions (CTC) and analyzes the correlation between the partition patterns and image structural features, selecting features based on their importance. In order to enable the model to effectively learn the rules of CU partitioning and accurately predict the optimal partitioning mode, it is necessary to establish a training dataset containing sufficient samples and labels. The second module extracts the features of the CU and the optimal partition pattern during VTM video coding and optimizes these features based on the structural features of the TT partition pattern, thereby reducing the complexity of feature extraction. Datasets for predicting the Horizontal Triple Tree (TT_H) and Vertical Triple Tree (TT_V) partition patterns are established. The final module uses these datasets to train and optimize the decision models. Subsequently, the trained models are embedded into the VTM 4.0. A threshold is set for the prediction results, and in the intra coding process, the decision on the TT partition is made based on this threshold.

In summary, the differences between the proposed method and existing methods can be summarized as shown in Table 1.

**Table 1:** The main differences and improvements.

Aspect	Gradient-based Methods	General ML-based Methods	Proposed Method
Feature Design	Handcrafted gradient/edge features; no statistical validation	Uses generic CU-level features; often not tailored to TT	Features selected based on statistical importance; texture–partition correlation explicitly analyzed
Dataset Construction	No training; heuristic rules only	Unified dataset for all partition types	TT-specific dataset designed for TT_H and TT_V with optimized texture features
Decision Mechanism	Rule-based thresholding	Model-based prediction but often external to VTM	Fully embedded in VTM 4.0 with adaptive threshold to skip RD calculations

### 2.1 Feature correlation analysis

**Fig 2:**
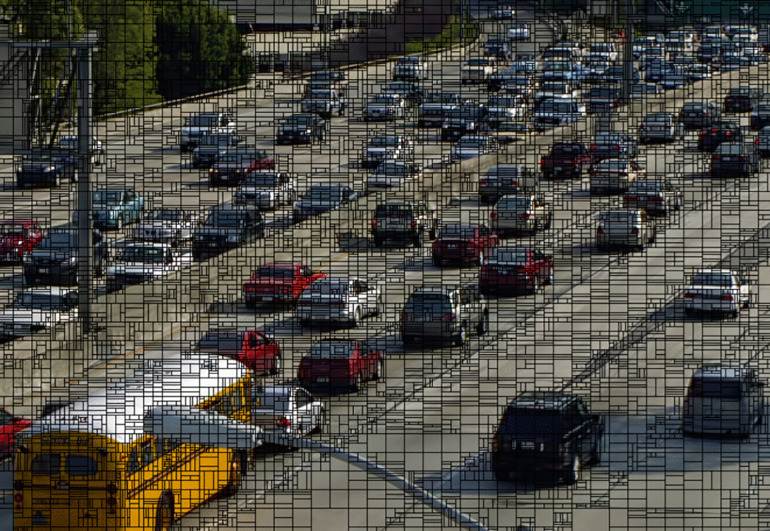
Visualization of the CU partitioning of the “Traffic” sequence encoded using VTM 4.0.

Fig 2 illustrates a visualization of the partition structure of the first frame of the “Traffic” video as it is coded using VTM 4.0. Observations of the video frame and its partition structure show that in areas with simple textures (such as roads and signs), the CU blocks are relatively large, whereas in areas with complex textures (such as vehicles and road markings), the CU blocks are relatively small. This indicates that the optimal partition pattern is closely related to the textural features of the video. Specifically, VVC tends to use larger square blocks to encode areas with simple and flat textures, and smaller rectangular blocks to encode areas with complex and uneven textures. This strategy ensures coding quality while achieving a maximal compression rate.

**Fig 3:**
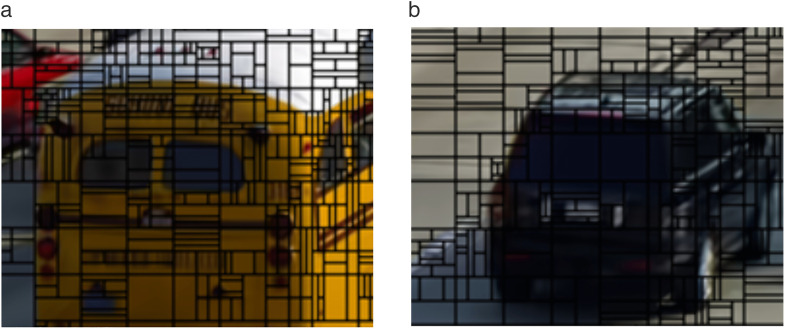
Partial visualization of CU partition structure.

Fig 3(a) and 3(b) illustrate local visualization of the CU partitioning using the VTM. Vertical partitions are used at the vertical edges of the vehicles, whereas horizontal partitions are employed at horizontal edges. These results suggest that the size, shape, and direction of the CU partition patterns can be predicted based on the textural features of the CUs. Specifically, horizontal partitions (such as BT_H or TT_H) are used at complex and horizontal edges, whereas vertical partitions (such as BT_V or TT_V) are used at complex and vertical edges.

**Fig 4:**
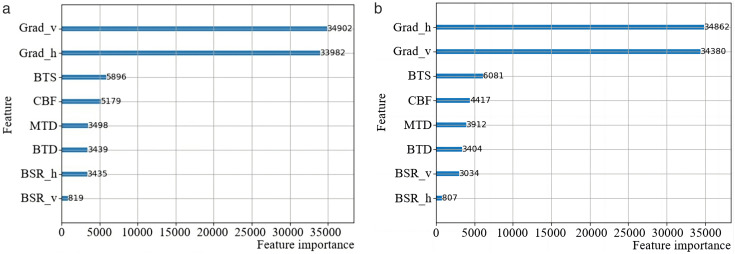
Feature importance when the optimal partition pattern is TT_H or TT_V. (a) Feature importance (TT_H). (b) Feature importance (TT_V).

To validate the correlation between the optimal CU partition pattern and texture features, certain CU features are extracted during the VTM coding process, and their importance is computed using LightGBM [22]. In contrast to approximate dynamic programming (ADP), which attains stable decision-making in dynamic systems through value-function approximation, the LightGBM leverages the structural characteristics of images to enable rapid partitioning decisions with greater interpretability and reduced implementation complexity [23]. Table 2 lists these features, including the texture features represented by the horizontal (Grad_h) and vertical gradients (Grad_v) of the luminance pixels. The larger the gradient value, the more pronounced the texture variation. In addition to the gradient features, we have incorporated five supplementary features inspired by the work of [21]. These features achieve high importance scores in the LightGBM analysis while adding relatively low complexity to feature extraction. Datasets are established, and the LightGBM models are trained. Finally, the importance of each feature is calculated based on the information gained, as illustrated in Fig 4. Notably, the importance of Grad_h and Grad_v is considerably higher than that of the other features, indicating a correlation between the textural features of the image and the optimal CU partition pattern. Based on these correlations, more efficient feature extraction strategies can be developed.

**Table 2:** Extracted CU features and their descriptions.

Feature	Description
Grad_h	The current CU luma pixel gradient in the horizontal direction.
Grad_v	The current CU luma pixel gradient in the vertical direction.
BSR_h	Block shape ratio: *BSR* = *lh*/(*lh* + *lw*) when divided by TT_H, *lh* indicates the CU’s height and *lw* indicates the CU’s width.
BSR_v	Block shape ratio: *BSR* = *lw*/(*lh* + *lw*) when divided by TT_V, *lh* indicates the CU’s height and *lw* indicates the CU’s width.
CBF	the residual can be considered to be 0, then CBF = 0, if there is a significant residual coefficient then CBF = 1.
BTS	BT’s superiority: taken as 0.5 when there are BTs with lower RD cost than other BTs, and 1 otherwise.
BTD	BT’s direction: when the optimal BT’s direction of two BTs on RD cost is the same as the TT’s direction to be tested, use 1; otherwise, use 0.
MTD	MTT’s depth: one-half of the depth of the BT or TT.

### 2.2 Feature extraction and optimization

**Fig 5:**
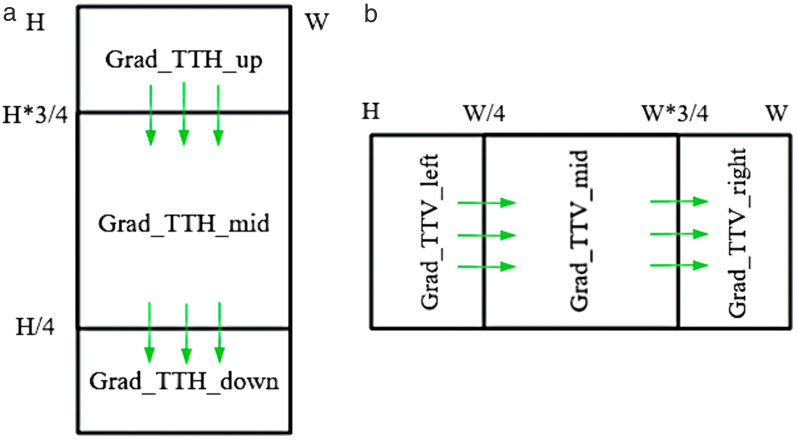
Gradients of the CU after TT_H (N × 2N CU) and TT_V (2N × N CU) partitioning. (a) TT_H. (b) TT_V.

There are various methods for describing texture features; however, their complexity increases the time required for feature extraction, without reducing the coding time. Since the luminance component varies with texture information, the gradient between luminance values serves as a viable representation of the texture information in video frames. High gradient values denote significant changes in texture information, while low gradient values imply smoother texture variations. Each TT partition forms three CU blocks, necessitating the separate calculation of texture gradients for each block. As depicted in Fig 5, in a TT_H partition, the three blocks are the upper block (Grad_TTH_up), middle block (Grad_TTH_mid), and lower block (Grad_TTH_down); in a TT_V partition, the three blocks are the left block (Grad_TTV_left), middle block (Grad_TTV_mid), and right block (Grad_TTV_right).

The texture features of each block include both the horizontal and vertical directions, totaling 12 gradients. Specifically, the horizontal gradient is computed as the sum of the differences between each pixel in the block and its left neighbor, while the vertical gradient is calculated as the sum of the differences between each pixel and the pixel directly below it. The formulas for calculating the texture features can be expressed as follows:


$$Grandh=\sum_{i=1}^{h}\sum_{j=1}^{w}\left|org(i+1,j)-org(i,j)\right|$$ (1)

$$Grandv=\sum_{i=1}^{h}\sum_{j=1}^{w}\left|org(i,j+1)-org(i,j)\right|$$ (2)

where *Gradh* denotes the horizontal gradient, *Gradv* denotes the vertical gradient, *h* denotes the height of the current CU, *w* denotes the width of the current CU, *i* and *h* denote the height boundaries of the partitioned CU, and *j* and *w* denote the width boundaries of the partitioned CU.

A simple representation of texture features involves the use of video pixel gradients. However, calculating the gradient features for each CU block using the aforementioned equations leads to considerable complexity; therefore, the feature equation needs optimization. The simplest and most effective optimization method is downsampling, which reduces the number of pixels by half-sampling both horizontally and vertically. According to Figs 4 and 5, when the CU is partitioned by the TT_H partition, the feature importance of the vertical gradient is higher. Consequently, horizontal gradients are downsampled by half. Conversely, when the CU block is divided by the TT_V partition, the feature importance of the horizontal gradient is higher, allowing for downsampling vertical gradients by half.

The average horizontal and vertical gradients of each block canbe expressed as follows:


Area_small_downsample_1/m = W*H/4/4,m =4,8 (3)

Area_large_downsample_1/m = W*H/4/2,m =4,8 (4)

where *Area_small_downsample_*1*/m* denotes the area of the smaller CU blocks after downsampling by 1/*m*, and *Area_large_downsample_*1*/m* denotes the area of the larger CU blocks after downsampling by 1/*m*.

As illustrated in Fig 5, when the width of the current CU is considerably smaller than its height, the optimal partition pattern of the CU tends to be horizontal; conversely, when the width of the CU significantly exceeds its height, a vertical partition is more favored. Consequently, in addition to the previously mentioned texture features based on pixel gradients within the CU block, the block shape ratio (*BSR*) is introduced to reflect the shape features of the current CU. The *BSR* is defined as the ratio of the block’s height or width to the sum of its height and width: for horizontal partitioning, which divides the block along its height, the ratio is calculated as the block’s height divided by the sum of its height and width; for vertical partitioning, which divides the block along its width, it is calculated as the block’s width divided by the sum of its height and width. Then, *BSR* can be represented as follows:


$$BSR=\frac{n}{H+W},\;\{\begin{array}{c} {}*20ln=H,\;TT\_Hpartition\\ n=W,\;TT\_Vpartition \end{array}$$ (5)

The average gradients and *BSR* are used as inputs for the dataset, with the optimal partition pattern of the CU serving as the label. The VTM 4.0 encoder is used to encode the first ten frames of 21 sequences from the CTC, and the average gradients, *BSR*, and corresponding optimal partition pattern are extracted for each CU block during the coding process. Two datasets are established to predict the TT_H and TT_V partition patterns. For the TT_H partition pattern, the input includes the average gradients (Gradh_TTH_up, Gradv_TTH_up, Gradh_TTH_mid, Gradv_TTH_mid, Gradh_TTH_down, Gradv_TTH_down) and *BSR*. If the optimal partition pattern is TT_H, the label is one; otherwise, it is zero. For the TT_V partition pattern, the input includes the average gradients (Gradh_TTV_left, Gradv_TTV_left, Gradh_TTV_mid, Gradv_TTV_mid, Gradh_TTV_right, Gradv_TTV_right) and *BSR*. If the optimal partition pattern is TT_V, the label is one; otherwise, it is zero.

The dataset constructed using the aforementioned method originally contains over one million entries. To reduce computational overhead and mitigate the impact of class imbalance on model training, a subset of approximately 200,000 entries is randomly sampled to form the final dataset. The labels within this dataset, denoted as 0 and 1, account for roughly 60% and 40% of the entries, respectively. The final dataset is then split into training, validation, and test sets in a ratio of 70%, 15%, and 15%, respectively.

### 2.3 Model training and embedding

The VTM evaluates all possible partition patterns of each Coding Tree Unit (CTU) from the bottom to the top. The proposed decision mechanism employs a LightGBM classifier to predict the partition pattern of the CU. The LightGBM classifier aggregates *N* weak classifiers (*f*_1_, *f*_2_, *f*_3_,..., *f*_*N*_) into a robust classifier model, represented as $F=\sum_{i=1}^{N}f_{i}$. These classifiers are utilized to predict the TT partition based on the features of the current CU. Two separate LightGBM classifiers are trained for the TT_H and TT_V partition patterns, and the prediction results inform the decision on whether the TT partitioning should be bypassed. This procedure aids in circumventing the computation of the RD cost for the partition pattern during the RDO process, thereby enhancing the coding speed.

**Fig 6:**
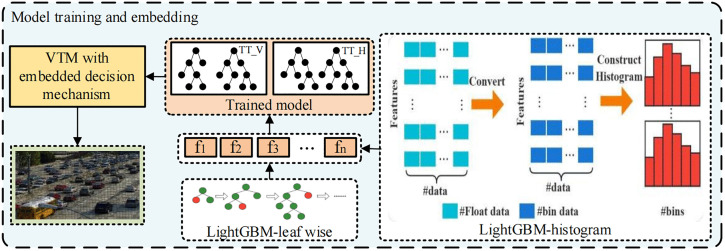
Model training.

As depicted in Fig 6, during the model training phase, the LightGBM classifier utilizes a histogram-based algorithm. This approach not only enhances computational efficiency but also serves as a form of regularization by substituting the original data with bins, where the number of bins determines the intensity of regularization. Given that the decision tree is a weak learner and regularization effectively mitigates model overfitting [22], the LightGBM classifier adopts a leaf-wise growth strategy, selecting the leaf with the highest split gain among all current leaves for splitting. Although the leaf-wise algorithm can reduce more errors with the same number of splits, it may result in a deeper decision tree structure. Consequently, to prevent overfitting and manage the tree’s complexity, the LightGBM classifier employs the *num_leaves* parameter to limit the maximum number of leaves. The relationship between *num_leaves* and *max_depth* is approximately *num_leaves* = 2^*max_depth*^, and the value of *num_leaves* should be less than 2^*max_depth*^.

The LightGBM classifier integrates gradient descent and boosting techniques through the introduction of various hyperparameters. During the training phase, hyperparameter optimization is essential to maximize the classifier’s performance. Initially, a higher learning rate is employed to expedite convergence. Subsequently, adjustments can be made to the fundamental parameters of the decision tree and the regularization parameters, followed by reducing the learning rate to improve the classifier’s accuracy. The final configurations of the model parameters are detailed in Table 3. Moreover, LightGBM controls overfitting by restricting the number of leaves (num_leaves) and employing several additional built-in regularization mechanisms. Specifically, min_data_in_leaf prevents the creation of leaves with too few samples, while lambda_l1 and lambda_l2 apply L1 and L2 regularization to the leaf weights, respectively. The training process also incorporates early stopping based on the validation loss, halting training if no improvement is observed over 50 consecutive iterations. These mechanisms collectively ensure that the model generalizes well and avoids overfitting.

**Table 3:** Model parameters.

Name	Boosting_type	Objective	Metric	Num_leaves	Learning_rate	Max_depth
Value	gbdt	Binary	auc	115	0.05	10

**Fig 7:**
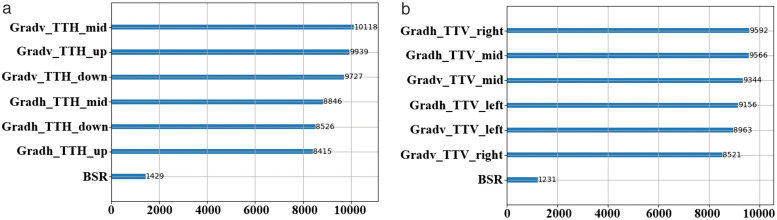
Feature importance of the LightGBM models. (a) Feature importance of the horizontal mode. (b) Feature importance of the vertical model.

As depicted in Fig 6, given that there are two directions in TT partitioning, two separate LightGBM models are trained using self-constructed datasets. The analysis of feature importance, as illustrated in Fig 7, reveals that within the horizontal-direction model, vertical gradient features hold greater importance than horizontal gradient features. Conversely, within the vertical-direction model, the importance of horizontal gradient features surpasses that of vertical gradient features. This finding aligns with the selection and optimization of partitioning pattern features. To reduce the video coding time, it is necessary to control the model prediction time while also enhancing its accuracy to ensure the coding quality. This requires a balance between the model’s complexity and accuracy. As a gradient-boosting model, the LightGBM classifier generates a new decision-maker with each training iteration. Consequently, reducing the number of training rounds can significantly reduce the model complexity. Ultimately, the accuracy of the horizontal-direction model reached 89.58%, whereas that of the vertical-direction model reached 89.45%.

In VVC, decisions regarding CU partition patterns depend on multiple constraints. For instance, all CTUs with a size of 128 × 128 split into 64 × 64 CUs using a Quad-Tree (QT) partition. From the 64 × 64 CU down to the 4 × 4 CU, six potential partition patterns exist: No Split, QT, Binary Tree Horizontal (BT_H), Binary Tree Vertical (BT_V), Ternary Tree Horizontal (TT_H), and Ternary Tree Vertical (TT_V). During the Rate-Distortion Optimization (RDO) process, the system explores all potential partition patterns from the bottom to the top, calculates their RD costs, and chooses the pattern with the lowest RD cost as the optimal partition pattern for the current CU. In the VTM, the calculation order follows QT, BT_H, BT_V, TT_H, and TT_V.

**Fig 8:**
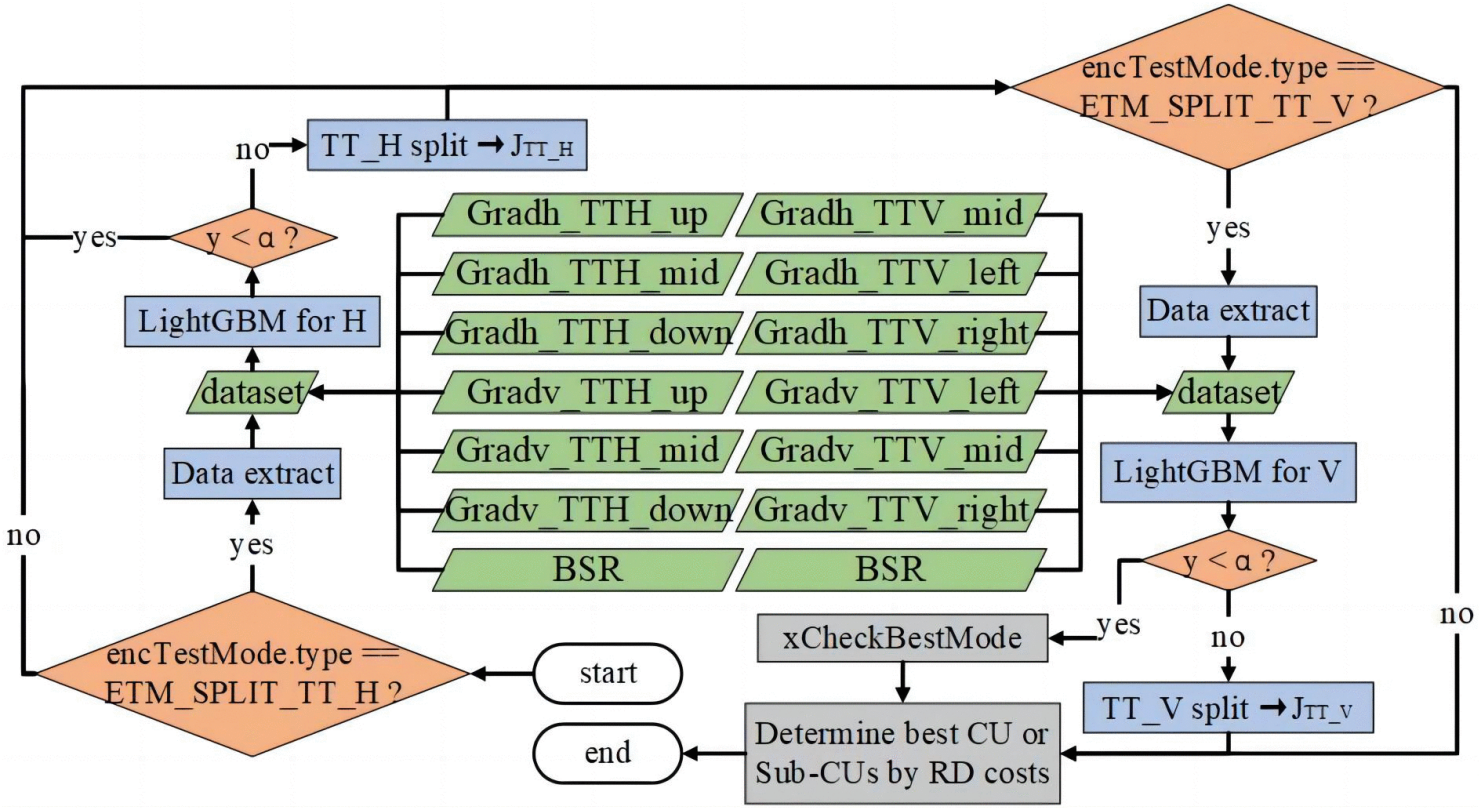
TT partition using the VTM embedded with a decision mechanism.

Fig 8 illustrates a flowchart of the decision mechanism embedded in the TT partitioning model. Before using the LightGBM classifier for prediction, seven features of the current CU are extracted. If the evaluated partition pattern is TT, the LightGBM classifier is used to assess the tested partition pattern. For a TT_H partition, the extracted features are input into the horizontal model specifically designed for predicting the TT_H partition to obtain a prediction value (*y*), which is then compared with the threshold *α* (*α* = 0.5). If *y* < *α*, the TT_H partition is bypassed, and the optimal partition pattern is selected based on the RD costs of the remaining patterns. If *y* ≥ *α*, the RD cost for the TT_H partition is calculated and compared with other partition patterns to ascertain the optimal one. Similarly, the features are input into the vertical model to predict the TT_V partition. If *y* < *α* (*α* = 0.5), the TT_V partition is bypassed; otherwise, the RD cost for the TT_V partition is computed and compared with other partition patterns.

## 3 Results and analysis

To ensure fairness and reproducibility in the experimental comparison, all experiments were conducted using the All-Intra configuration, consistent with the original VTM 4.0 settings. Importantly, both the proposed model and the LNN model [21] were trained and tested on the same dataset, and all experiments were performed on the same hardware platform and under identical QP settings, enabling a direct and fair comparison between the two models. All experiments were conducted on a Windows 10 Professional 64-bit computer with an Intel Core i7-9700 CPU @ 3.00GHz and 8GB of RAM.

The Bjøntegaard delta peak signal-to-noise rate (BD-PSNR), BDBR, time-saving (TS), and the TS/BDBR ratio serve to evaluate coding quality and efficiency. BD-PSNR quantifies the difference in *PSNR*_*Y*_ between two methods at an equivalent bit rate; a positive value indicates an enhancement in coding quality by the optimized algorithm. Conversely, BDBR measures the rate savings between two methods at the same objective quality, with a negative value signifying improved coding performance by the optimized algorithm. Furthermore, *TS*_*LGBM*_ denotes the percentage of coding time saved by the decision mechanism relative to VTM 4.0, while *TS*_*LNN*_ represents the percentage of coding time saved by the LNN algorithm as described in [21] compared to VTM 4.0. The TS/BDBR ratio reflects the combined effect of reducing both time and bit rate. The calculations for *TS*_*LGBM*_ and *TS*_*LNN*_ appear as follows:


$$TS_{LGBM}=\frac{\text{1}}{\text{4}}\sum_{QP_{i}\in \{22,27,32,37\}}\frac{T_{VTM}(QP_{i})-T_{LGBM}(QP_{i})}{T_{VTM}(QP_{i})}\times 100\%$$ (6)

$$TS_{LNN}=\frac{\text{1}}{\text{4}}\sum_{QP_{i}\in \{22,27,32,37\}}\frac{T_{VTM}(QP_{i})-T_{LNN}(QP_{i})}{T_{VTM}(QP_{i})}\times 100\%$$ (7)

where *T*_*VTM*_ denotes the time required for coding using the VTM 4.0, *T*_*LNN*_ denotes the time required for coding using the LNN algorithm, *T*_*LGBM*_ denotes the time required for coding using the LightGBM algorithm described in this paper, and *QP*_*i*_ denotes for the different QP values used during the coding. Table 4 lists the all-intra experimental results, with “Class” for the video categories and “Sequence” for the CTC sequences. *BDBR*_*LGBM*_ and *BDBR*_*LNN*_ denote *BDBR*_*YUV*_ changes; *BD-PSNR*_*LGBM*_ and *BD-PSNR*_*LNN*_ denote *BD-PSNR*_*Y*_ changes, and *TS*_*LGBM*_ and *TS*_*LNN*_ denote coding time savings, and Average denotes the mean values, using the LightGBM and LNN algorithms versus the VTM 4.0 model, respectively.

**Table 4:** Experimental results comparing the proposed decision mechanism and the LNN algorithm using the VTM 4.0.

Class	Sequence	*BDBR*_*LNN*_	*BD*-*PSNR*_*LNN*_	*TS*_*LNN*_	*TS*_*LNN*_/ *BDBR*_*LNN*_	*BDBR*_*LGBM*_	*BD*-*PSNR*_*LGBM*_	*TS*_*LGBM*_	*TS*_*LGBM*_ */* *BDBR*_*LGBM*_
A1	Campfire	0.79%	−0.02%	42.73%	54.09	0.63%	−0.02%	46.38%	73.62
	FoodMarket4	0.61%	−0.02%	45.79%	75.07	0.43%	−0.02%	42.02%	97.72
	Tango2	0.62%	−0.01%	44.12%	71.16	0.48%	−0.01%	43.68%	91
A2	CatRobot	1.18%	−0.03%	44.45%	37.67	0.98%	−0.03%	46.12%	47.06
	DaylightRoad2	1.08%	−0.03%	47.63%	44.1	0.93%	−0.02%	43.29%	46.55
	ParkRunning3	0.51%	−0.03%	37.91%	74.33	0.32%	−0.02%	42.41%	132.53
ADD	PeopleOnStreet	1.50%	−0.08%	48.73%	32.49	0.96%	−0.05%	43.18%	44.98
	Kimono	0.58%	−0.02%	48.84%	84.21	0.46%	−0.02%	52.93%	115.07
	Traffic	1.47%	−0.08%	45.02%	30.63	1.03%	−0.05%	39.09%	37.95
B	BasketballDrive	1.28%	−0.04%	46.65%	36.45	0.81%	−0.03%	46.33%	57.2
	BQTerrace	1.19%	−0.05%	42.13%	35.4	0.74%	−0.03%	35.68%	48.22
	Cactus	1.10%	−0.04%	44.61%	40.55	0.83%	−0.03%	42.88%	51.66
	MarketPlace	0.63%	−0.03%	48.12%	76.38	0.46%	−0.02%	52.45%	114.02
	RitualDance	1.14%	−0.06%	47.45%	41.62	0.83%	−0.05%	49.66%	59.83
C	BasketballDrill	1.86%	−0.10%	45.17%	24.28	1.41%	−0.07%	45.74%	32.44
	BQMall	1.46%	−0.09%	46.87%	32.1	0.92%	−0.06%	43.64%	47.43
	PartyScene	0.78%	−0.06%	44.00%	56.41	0.46%	−0.03%	37.10%	80.65
D	RaceHorses	0.84%	−0.06%	42.35%	50.42	0.52%	−0.04%	44.77%	86.1
E	FourPeople	1.63%	−0.10%	47.71%	29.27	1.23%	−0.07%	45.15%	36.71
	KristenAndSara	1.39%	−0.07%	46.09%	33.16	0.94%	−0.05%	43.26%	46.02
	Johnny	1.61%	−0.07%	46.88%	29.12	1.21%	−0.05%	46.22%	38.2
	Average	1.11%	−0.05%	45.39%	47.09	0.79%	−0.03%	44.38%	65.95

Table 4 reveals the performance patterns across different video categories. Sequences characterized by high texture complexity and motion, such as category A2 including ‘CatRobot’ and ‘DaylightRoad2’ and category E including ‘FourPeople’, tend to exhibit slightly higher BDBR increases of approximately 0.9% to 1.2% while still achieving substantial time savings of around 43% to 46%. This behavior is expected, as complex textures pose more challenging prediction tasks. In contrast, sequences dominated by smooth regions, such as category A1 including ‘Tango2’ and ‘FoodMarket4’, benefit from a highly favorable trade-off, with BDBR increases of only 0.43% to 0.63% and time savings of approximately 42% to 46%. Notably, compared with LNN, the proposed method consistently achieves higher TS/BDBR ratios across all categories, highlighting its superior overall efficiency.

From Table 4, it is evident that using the decision mechanism, the coding time was reduced by an average of 44.38% compared to the original VTM 4.0, with the BDBR increasing by only 0.79% and the BD-PSNR decreasing by 0.03%. The performance in terms of the BDBR and BD-PSNR proved to be superior to that of the LNN algorithm, and the TS/BDBR value was considerably higher than that of the LNN algorithm under similar time-saving conditions. These data indicate that the proposed decision mechanism could reduce coding time while maintaining coding quality.

Additionally, a complexity analysis was conducted. During the inference phase, the proposed method requires approximately 60 MB of memory to store the ensemble of decision trees, and the inference for a single video sequence involves roughly 2 GFLOPs, which is negligible compared with the full VVC encoding pipeline. Consequently, the proposed approach provides a lightweight and efficient solution, achieving a favorable trade-off between prediction accuracy and encoding complexity.

## 4 Conclusion

A fast decision mechanism was proposed for the TT partition in VVC intra coding, which skips the RD cost calculation process for the TT partition. This solved the problems of high complexity and time consumption in CU partitioning during VVC intra coding. Initially, the correlation between the partition patterns and image structural features is analyzed, and the importance of video features was calculated, and texture features were selected to predict the optimal partition pattern. Subsequently, gradients were used to efficiently represent the video texture features. The features were optimized based on the structural features of the CU partitioned by the TT partition pattern, and datasets for the TT_H and TT_V partition patterns were established. Finally, these datasets were used to train the LightGBM classifier, which was then embedded in VTM 4.0 to predict the partition patterns. By skipping the calculation process of the RD cost for the TT partition based on the prediction results, the time required for VVC intra coding was reduced, thereby enhancing the coding efficiency. Experiments demonstrated that the coding efficiency of the proposed decision mechanism was superior to that of the VTM 4.0.

In future studies, more effective methods will be investigated to represent texture information, and additional features related to partition patterns will be explored to further enhance the accuracy and efficiency of partition pattern prediction. It should be noted that LightGBM is sensitive to the predefined threshold *α*, and identifying an optimal, potentially adaptive threshold for different content types requires further investigation. Moreover, extending this framework to inter-frame coding represents a promising direction for future work.
